# In vivo measurement of shear modulus of the human cornea using optical coherence elastography

**DOI:** 10.1038/s41598-020-74383-4

**Published:** 2020-10-15

**Authors:** Antoine Ramier, Amira M. Eltony, YiTong Chen, Fatima Clouser, Judith S. Birkenfeld, Amy Watts, Seok-Hyun Yun

**Affiliations:** 1 Wellman Center for Photomedicine and Harvard Medical School, Massachusetts General Hospital, 50 Blossom St., Boston, MA USA; 2 Harvard-MIT Division of Health Sciences and Technology, Cambridge, MA USA; 3 Department of Automation, Tsinghua University, Beijing, 100084 China; 4 Research Laboratory of Electronics, Massachusetts Institute of Technology, 77 Massachusetts Ave, Cambridge, MA 02139 USA; 5 Instituto de Optica (IO-CSIC), C/Serrano, 121 Madrid, Spain; 6 Department of Ophthalmology, Massachusetts Eye and Ear, 243 Charles Street, Boston, MA 02114 USA

**Keywords:** Biophotonics, Optical imaging, Rheology

## Abstract

Corneal stiffness plays a critical role in shaping the cornea with respect to intraocular pressure and physical interventions. However, it remains difficult to measure the mechanical properties noninvasively. Here, we report the first measurement of shear modulus in human corneas in vivo using optical coherence elastography (OCE) based on surface elastic waves. In a pilot study of 12 healthy subjects aged between 25 and 67, the Rayleigh-wave speed was 7.86 ± 0.75 m/s, corresponding to a shear modulus of 72 ± 14 kPa. Our data reveal two unexpected trends: no correlation was found between the wave speed and IOP between 13–18 mmHg, and shear modulus decreases with age (− 0.32 ± 0.17 m/s per decade). We propose that shear stiffness is governed by the interfibrillar matrix, whereas tensile strength is dominated by collagen fibrils. Rayleigh-wave OCE may prove useful for clinical diagnosis, refractive surgeries, and treatment monitoring.

## Introduction

The cornea plays a major role in human vision by providing approximately two thirds of the refractive power of the eye. The meniscus shape of the cornea results from a mechanical equilibrium of the cornea with respect to intraocular pressure (IOP). A change in the mechanical homeostasis can alter the corneal shape and thereby affect visual acuity. This relationship is also the basis for vision-improving refractive surgeries, such as laser-assisted in-situ keratomileusis (LASIK) and limbal relaxing incision (LRI), wherein refractive errors are corrected by a combination of tissue ablation and the resulting remodelling driven by tension and stiffness. Corneal tissues are thought to lose stiffness in degenerative corneal disorders, such as keratoconus, which leads to vision-impairing conical ectasia^1,2^. An accepted treatment for ectasia, corneal crosslinking (CXL), aims to mechanically stabilize the cornea^3^. Therefore, measurement of the mechanical parameters of the cornea is useful in assessing corneal health, improving refractive treatments, and diagnosing degenerative disorders^4,5^.

While the measurement of IOP by tonometry is well established and routinely performed as part of standard care, it remains a challenge to characterize the physical integrity of the cornea quantitatively in a clinical setting. Standard mechanical characterization techniques, such as strip extensometry, compression and inflation tests, have provided basic understanding of corneal biomechanics ex vivo, but these invasive methods are not applicable or undesirable for clinical use^6,7^. For in vivo measurement, elastography holds promise, and a number of specific approaches have been devised with different advantages. Strain-imaging ultrasound elastography and shear-wave elasticity imaging (SWEI) have been used to examine liver sclerosis and cancers, but their spatial resolution and required acoustic energy are not adequate for routine corneal applications^8,9^. Surface wave elastometry using a pair of ultrasound transducers was proposed for corneal applications, but the two-point approach offered no spatial resolution or ability to distinguish different types of elastic waves^10^. Ocular response analysers and optical elastography using air-puff stimuli provide empirical indices related to the viscoelastic properties of the cornea^11–13^. However, these approaches do not offer a quantitative readout of elastic modulus and require complex numerical analysis for spatially-resolved measurements^14^. Brillouin microscopy can measure longitudinal modulus with high spatial resolution^15,16^, but it is shear and Young’s moduli that are directly related to corneal stiffness with respect to external force. Optical coherence elastography (OCE) has emerged as a promising technique with high spatial resolution and high sensitivity to mechanical deformation of tissue^17,18^. Recently, a compression-based OCE technique has been tested in human subjects^19,20^ but the ability to measure elastic modulus quantitatively remains to be developed. Quantitative approaches based on the excitation of elastic waves have been widely studied with ex vivo tissues and live animals^21–24^.

Here, we report, for the first time to our knowledge, in vivo quantitative measurement of the shear modulus of the human cornea. This measurement was made possible by employing an OCE system^25,26^ with a miniature contact probe that excites low-energy elastic waves safely on the human cornea over a frequency range centred around 10 kHz. This relatively high excitation frequency induces Rayleigh-type elastic waves in the human cornea, enabling measurement with high accuracy and spatial resolution. We also demonstrate in vivo measurement of the human sclera. A pilot study of 12 healthy subjects yielded interesting findings that were unexpected from previous ex vivo results^27^, such as the dependence of shear modulus on age and physiological IOP levels. We provide possible explanations for our observation based on a constitutive mechanical model of the cornea.

## Results

### Mechanical model of the cornea

**Figure 1:**
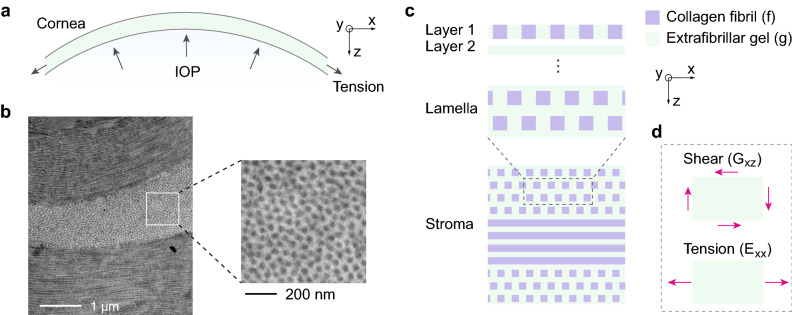
Corneal microstructure and constitutive model. (**a**) Corneal anatomy and tensile stress resulting from intraocular pressure. (**b**) Electron micrograph of a porcine cornea. (**c**) Microstructural model of the corneal stroma comprising lamellae. (**d**) Schematic of external shear and tensile forces (arrows) corresponding to (respectively) out-of-plane shear modulus ($G_{xz}$) and in-plane Young’s modulus ($E_{xx}$).

The corneal stroma is responsible for 90% of the corneal thickness and provides the largest part of its mechanical strength and resistance to IOP (Fig. 1a). The stroma is a collagen and proteoglycan-rich connective tissue. The collagen molecules are arranged in fibrils with a diameter of about 30 nm. The fibrils form a lamellar structure^28^, as shown in Fig. 1b. Within each lamella, the fibrils are organized into a hexagonal lattice that is cross-linked within a hydrated proteoglycan-rich matrix^29^. A single lamella is about 1.5–3 µm thick and is oriented parallel to the corneal surface. The human stroma is composed of about 200 lamellae, which are stacked on top of each other with fibril orientation of adjacent layers crossing at large angles. The preferential orientation of lamellae and their degree of interweaving vary with depth and location in the stroma. This complex microarchitecture gives rise to the macroscopic-scale mechanical properties of the cornea.

To understand the mechanical properties and elastic-wave propagation within the cornea, we modelled the corneal stroma as a two-component constitutive structure comprising collagen fibrils (*f*) and extrafibrillar hydrogel matrix (*g*). The elastic moduli of an individual lamella can be obtained with good accuracy by assuming square-shaped fibres with the correct volume ratio and using well-established averaging rules^30,31^. Briefly, the compliance matrices of the fibril-containing layer (layer 1; Fig. 1c) and extrafibrillar layer (layer 2) are computed. These repeating units are then stacked to form a single lamella. The last step is to stack lamellae with different fibre orientations. This calculation is described in the Supplementary Materials. The main conclusion of this analysis is that the overall shear modulus of the stroma is governed by the shear modulus of the extrafibrillar matrix. The contribution of the much stiffer collagen fibrils is quenched by the surrounding softer medium. When the extrafibrillar space is assumed to be isotropic with shear modulus $\mu_{g}$, the complete lamellar structure gives rise to a transverse isotropic symmetry. The shear modulus $G_{ij}$ of the cornea is calculated to be $\sim 1.6 \mu_{g}$ for out-of-plane shear ($G_{zx}$, $G_{zy} \equiv G$) and $\sim 1.9 \mu_{g}$ for in-plane shear $G_{xy}$.

We note that conventional tensile measurement or extensometry of corneal tissues determines Young’s modulus (*E*) in the direction along the plane (Fig. 1d). In a structure of randomly oriented lamellae, our model yields in-plane Young’s modulus, $E_{xx} /3 \approx \sim 1.9 \mu_{g}$ (Supplementary Materials). However, if the lamellar orientation is not completely random, but instead has a dominant direction along the x-axis, $E_{xx}$ could reflect the unquenched contribution of collagen fibrils (Supplementary Materials).

With the modest degree of anisotropy, the propagation speed of shear waves is direction dependent. Horizontal shear waves propagating along the optical axis (z-axis) have a speed of $(G_{xy} /\rho )^{0.5}$, where $\rho$ is mass density. Elastic waves guided along the corneal plane are described as Lamb waves with frequency-dependent speed^21^ (Supplementary Fig. S1). A mechanical stimulus comprising oscillatory normal stress can excite elastic waves that propagate as guided modes in the cornea. The relative amplitudes of the individual modes can be calculated using overlap integrals of the displacement (or stress) profiles of the modes and the mechanical stimulus (Supplementary Fig. S1). The effective propagation speed of the excited modes at frequencies above ~ 6 kHz is nearly a constant approaching the Rayleigh surface wave limit at the air-cornea interface. This plateau region offers a good window for the measurement of shear modulus because it is less sensitive to the excitation frequency and to morphological factors such as the corneal thickness. The Rayleigh wave speed is given by $c_{R} \approx 0.95 c_{s}$. Therefore, the shear modulus of the corneal tissue is determined using the following equation:

1 $$G \approx 1.1 \rho c_{R}^{2}$$

### OCE measurement

**Figure 2:**
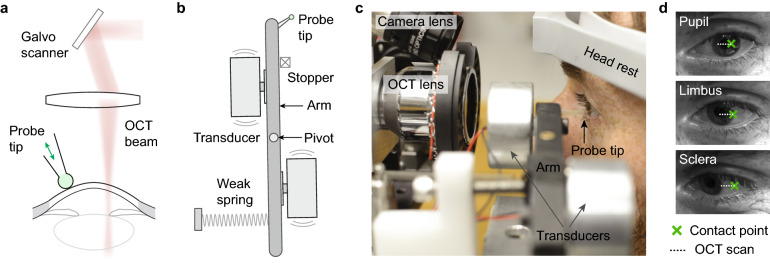
The OCE system. (**a**) Schematic of the corneal OCE technique using a vibrational contact probe. (**b**) Schematic of the contact probe. The probe tip is mounted on a lever arm which is driven by a pair of acoustic transducers. The lever arm complies with subject motion by rotating around a pivot axis. (**c**) Picture of the OCE prototype and a human subject. (**d**) Monitoring camera view of the eye for three different measurement locations in the central cornea, limbus, and sclera, respectively. The contact point of the probe is marked by a green cross, and the OCT-beam scan path is shown as a dotted line.

For human measurements, we devised a contact probe that can safely excite Rayleigh-type elastic waves in the cornea and allow us to measure the amplitude and speed of elastic waves using an OCT system (Fig. 2a). The probe is comprised of a pair of large-area acoustic transducers that are operated in a push–pull configuration. The vibration is transferred to a probe tip with a diameter of 1 mm through a mechanical arm (Fig. 2b). The control signal for the transducers was synchronized with beam scanning and data acquisition^25,26^. A topical anaesthetic was administered to the eye prior to applying the contact probe, and the procedure was well tolerated by the subjects. The probe is spring-loaded so that it maintains a constant force of about 20 mN when the tip contacts the corneal surface (Fig. 2c). Applying the Hertz model of elastic contact, we estimate that the contact radius the probe tip makes with the cornea is 0.38 mm, and the indentation of the cornea after contact is about 0.15 mm, comparable with established tonometry and pachymetry protocols. This relatively gentle contact pressure ensures an efficient excitation of elastic waves while posing minimal risk to the corneal surface. Greater contact force should be avoided as it may cause discomfort to the subject due to noticeable corneal deformation and will increase the risk of friction-induced damage to the corneal surface in case of head movements.

For each human subject, OCE measurement was performed in the left eye at 3 different points: central cornea, peripheral cornea, and sclera, respectively. The contact was established by moving the probe gently towards the cornea until a restoring spring was engaged, limiting the contact force. For central cornea measurements, the contact point was ~ 1 mm away from the pupil centre towards the lateral side (away from the nose); for peripheral corneal measurement, the contact point was at the limbus, and for scleral measurement, the contact point was located at an offset of ~ 5 mm laterally from the scleral-corneal boundary (Fig. 2d). All contact points were located close to the horizontal meridian of the eye. The vibration amplitude produced by the probe was about 1 µm at the corneal surface. The frequency of vibration was continuously step-tuned from 2 to 16 kHz with an interval of 2 kHz and a duration of 0.125 s per frequency (so a single scan took 1 s), and the frequency ramp was repeated up to 10 times (10 s) while the OCT beam was scanned along the horizontal axis from the contact point (Supplementary Fig. S2). The maximum possible vibration frequency was limited to 16 kHz for the mechanical actuator we used. The wavelength and propagation distance of an elastic wave decrease with increasing excitation frequency. Therefore, the step size and span of the beam scan were varied according to the excitation frequency in order to optimize measurement accuracy and minimize scan duration. For data fidelity, we performed 3–4 separate measurements in the same region, each time retracting the probe tip from the eye and repositioning to the same location as closely as possible. The imaging session for each subject took about 15 min including short intermissions between measurements. More detail of the system operation is described in “Methods”.

We estimate that the maximum acoustic intensity in the cornea is 7.5 mW/cm^2^, well below the spatial-peak temporal-average intensity limit of 17 mW/cm^2^ for ophthalmic ultrasound^32^. To confirm the safety of the mechanical stimulus, we performed fluorescein eye staining within 15–45 min after OCE measurement to examine potential corneal surface damage. 9 of 12 subjects exhibited no dye stain (grade 0) or trace dye mark (grade 1), and 3 subjects showed small staining spots, indicating minor reversible epithelial disruption. Corneas of all subjects were assessed by an ophthalmologist and considered unharmed. Based on this result, the OCE technique using the contact probe is deemed safe.

**Figure 3:**
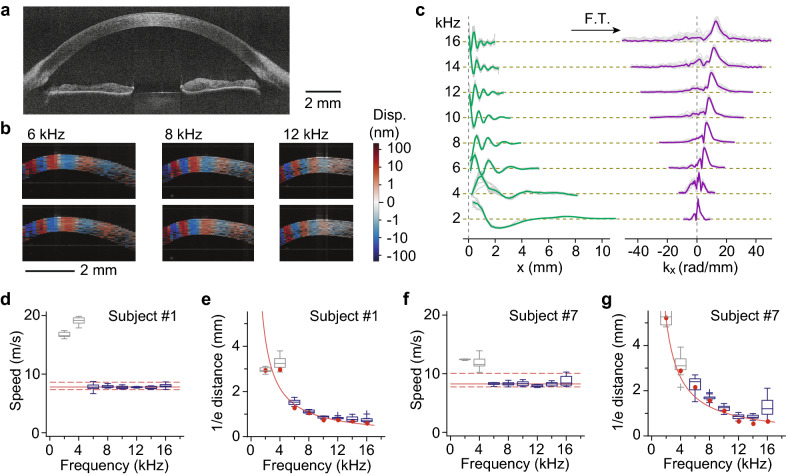
OCE of the human cornea in vivo. (**a**) Cross-sectional OCT image. (**b**) Vibrography images of elastic waves excited at different frequencies. Colour represents the displacement velocity (red: up, blue: down). (**c**) (Left) Displacement waveforms measured at the corneal surface as a function of the propagation distance and (Right) the corresponding spatial frequency representation obtained by the Fourier transform. Solid lines represent the average trace of four measurements. (**d**–**g**) Results obtained from two human subjects. The wave speed (**d**) and 1/e propagation distance (**e**) of Subject #1 (30-year old male) at the corneal centre. The wave speed (**f**) and 1/e propagation distance (**g**) at the corneal centre of Subject #7 (66-year old male). The box-whisker data representation corresponds to the quartiles and min–max of 10 frequency scans. Solid lines (red) represent the median (**d**,**f**) and curve fits to the data (**e**,**g**) over a range of 6–16 kHz. Dashed lines in (**d**) and (**f**) indicate 95% central quantile intervals. Greyed-out boxes correspond to frequencies that were left out of the analysis due to interference with spurious waves or insufficient wave amplitude.

Figure 3a,b show OCE images obtained from a healthy human subject. The cross-sectional vibrography images reveal the vertical displacement profiles of the elastic waves excited at different frequencies (Fig. 3b). The displacement patterns are uniform throughout the corneal thickness, suggesting shear-like waves. The displacement profiles over the $x$ coordinate and time $t$ at the cornea–air interface were extracted from the dataset. To remove contributions from spurious elastic waves and extract the Rayleigh-type elastic wave, we performed a 2-dimensional Fourier transform of the displacement profiles, moving the data from the $x$ domain to the wavenumber $k_{x}$ domain and from *t* to frequency *f*. Then, a narrowband frequency filter centred at the driving frequency was applied to obtain purified waveforms and determine the peak wavenumber for each frequency (Fig. 3c), from which the attenuation and speed of the waves were computed.

To account for the corneal curvature, we express the propagation distance, instead of $x$, in terms of a curvilinear coordinate, $s = \smallint ds$, along the corneal surface, where $ds^{2} = dx^{2} + dy^{2} + dz^{2}$ and the integral path follows the curved corneal-air interface. After a 2D Fourier transform from $s$ to wavenumber $k_{s}$, the peak wavenumber $k_{s}$ is determined, and the surface-wave velocity $c(f) = 2\pi fk_{s}$ is measured. The attenuation coefficient was calculated by fitting a linear function to the natural logarithm of the displacement magnitude. The 1/*e* propagation distance is determined from the reciprocal of the attenuation coefficient.

OCE measurements were performed on a total of 12 healthy subjects. The individual plots of wave velocity and attenuation length versus frequency for central corneas are displayed in Supplementary Figs. S3 and S4. Figure 3d–g show representative data obtained from two subjects of different ages (#1, 30 year-old male; and #7, 66 year-old male). The speed measurements at 2 and 4 kHz were often erroneous due to the interference of spurious waves traveling at much faster speeds and those reflected back from the corneal-scleral boundaries, as previously observed in porcine eyes^26^, so these low-frequency data were ignored in later processing steps. We also noted that the wave amplitude at higher frequencies > 12 kHz was sometimes insufficient to accurately extract the wave velocity for some subjects, presumably due to inconsistent contact between the vibrating tip and the corneal surface, and these low-signal data were also ignored in curve fitting to determine the speed and attenuation. The average wave speed value obtained from 12 subjects at the pupil centre was 7.86 ± 0.75 m/s (mean ± standard deviation). Since the bulk mechanical properties of the cornea are unlikely to vary considerably within the small region at the corneal centre, the deviation is thought to occur due to inconsistency in contact pressure, which can be improved. From Eq. (1), the measured wave speed corresponds to a shear modulus, $G$ = 72 ± 13.7 kPa. The 1/*e* propagation distance decreases with frequency, with a 1/$f$ dependence. The attenuation coefficients were 0.12 ± 0.010/mm/kHz for Subject #1, 0.11 ± 0.006/mm/kHz for Subject #7, and ~ 0.11 ± 0.014/mm/kHz on average for the 12 subjects.

**Figure 4:**
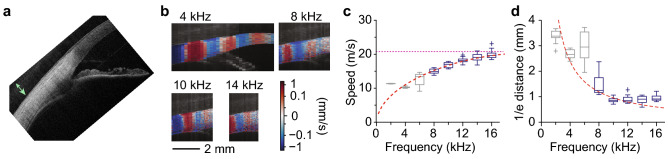
OCE of the human sclera in vivo. (**a**) Standard cross-sectional OCT image of a subject’s sclera and peripheral cornea. Arrow (green) indicates the approximate contact point of the excitation probe. (**b**) Vibrography images of the elastic waves excited at different frequencies. Colour represents the displacement velocity (red: up, blue: down). (**c**) Wave speed at the posterior sclera as a function of frequency. Dashed lines (red): theoretical curve fit based on $c_{s}$ = 22 m/s and $h$ = 610 µm. Dotted line (magenta): asymptotic value for the Rayleigh wave speed. (**d**) The 1/e propagation distance. Dashed line (red): curve fit.

Figure 4 depicts representative OCE data obtained from the anterior sclera of a healthy subject. The vibrography maps show uniform shear-like displacements (Fig. 4b). The wavelength is longer in the sclera than in the cornea by a factor 2. The dispersion curve exhibits a frequency dependence characteristic of Lamb waves (Fig. 4c). A theoretical curve based on the Lamb wave model fits the measured data reasonably well. Dispersion might also arise from the frequency-dependent measurement range, which covers a wider area at low frequencies and thus be more biased by gradients of tissue stiffness across the corneoscleral boundary. In both cases, the high-frequency limit provides a good estimate of the scleral Rayleigh-wave velocity. The shear modulus extracted from the data is ~ 510 kPa. The 1/e distance is ~ 1 mm, similar to that measured in the cornea (Fig. 4d), corresponding to 3 × higher attenuation coefficient in the sclera when the 3 × longer wavelength is considered. The higher attenuation may be due to increased elastic scattering of the waves due to greater inhomogeneity of scleral tissue compared to corneal tissue. The strong attenuation makes scleral OCE more challenging than corneal measurement.

**Figure 5:**
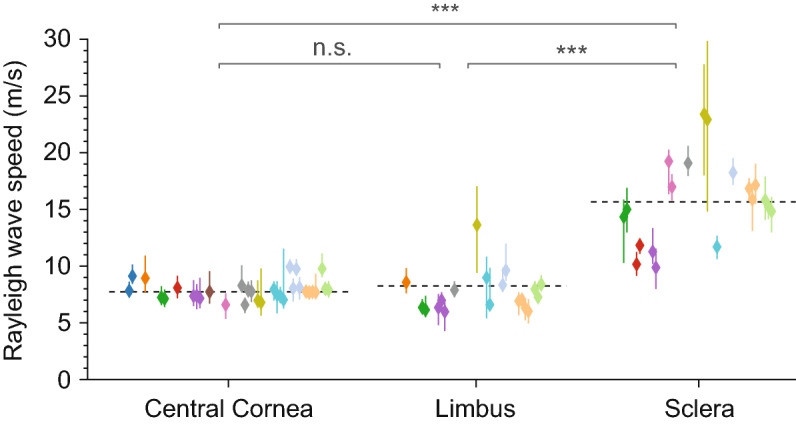
Rayleigh-wave speeds measured at different regions in the anterior surface of the eye in vivo at frequencies of 6–10 kHz. Colour indicates different human subjects. Multiple data points of the same colour correspond to repeated measurements. *ns* non-significant, ***p < 0.001. Dashed line: average level at 7.85 m/s.

The mean wave speed measured at the sclera from the 12 subjects was 15.7 ± 4.0 m/s (Fig. 5). This corresponds to a shear modulus of 310 ± 150 kPa. The higher modulus for the sclera compared to the cornea is compatible with the thicker and denser collagen fibrils in the sclera and is consistent with previous ex vivo measurements^33^. The large variation among subjects indicates that there is relatively large heterogeneity of the scleral tissues, and possibly some remaining waveguiding effect due to the longer elastic wavelength in the sclera.

### Correlation of shear modulus with age and IOP

The wave speed at the peripheral cornea was similar to the central cornea except for one subject (#9; olive-coloured) (Fig. 5). The mean Rayleigh-wave speed at the limbus was 8.23 ± 0.9 m/s including the outlier, and 7.58 ± 1.09 m/s excluding the outlier, which in both cases are not statistically significantly different from the values at the pupil centres.

**Figure 6:**
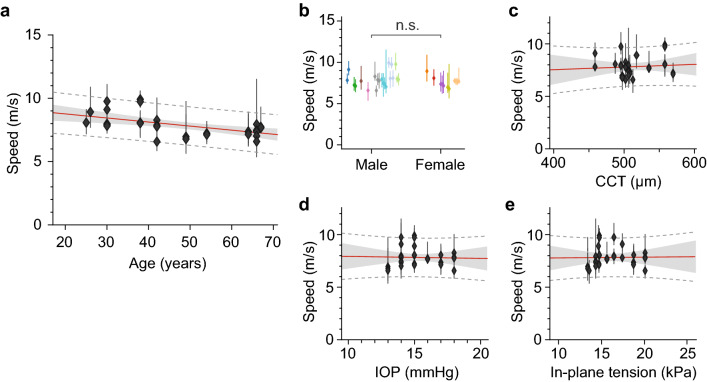
Dependence of the wave speed measured in the central cornea on various physiological factors. (**a**) Age, (**b**) gender, (**c**) CCT, (**d**) IOP, and (**e**) in-plane tension. Points and error bars represent the median and the central quantile range, respectively. Red line: best linear regression fit; shaded grey area: confidence interval (95%); dotted line: prediction interval.

We analysed the human data using linear regression to examine the correlation of corneal shear modulus with various physiological parameters. The wave speed was found to decrease with age, with a slope of − 0.32 (± 0.17) m/s per decade (95% confidence interval) (Fig. 6a). Assuming the same corneal mass density of 1.05 g/cm^3^, the out-of-plane shear modulus can be expressed as $G \approx 98 - 0.58 x$ (kPa), where $x$ denotes age in years. The age-related *decrease* of shear modulus, by ~ 8% per decade, is rather surprising given the evidence showing age-related *increase* of tensile Young’s modulus by ~ 8% per decade^27^. We will discuss this observation in the next section.

No correlation was found between corneal shear modulus and gender (Fig. 6b) or central corneal thickness (CCT) (Fig. 6c). We also found no dependence on IOP (Fig. 6d) or in-plane tension calculated from the IOP (Fig. 6e). The lack of correlation with IOP and IOP-induced tension was also rather interesting since we expected a positive correlation with IOP. We will also discuss this finding in the next section.

## Discussion

We have shown that Rayleigh-wave OCE is a safe and effective technique for quantifying the shear properties of the human cornea in vivo. It allows for spatially resolved measurement in close proximity to a given contact point of the probe. The shear modulus of the cornea measured over a frequency range of 6–16 kHz in healthy subjects (ages 25–65 years) was 68 kPa, with a 95% confidence interval of 59–75 kPa. A handful of studies have attempted to measure the shear modulus of corneal tissues ex vivo using standard tensiometer tests. Reported values vary widely from 2.0 kPa^34^ to 80 kPa^35^. This large range may be partly due to variation in the hydration level of the ex vivo samples tested, and also partly due to variation in the mechanical bias point given the high nonlinearity of corneal elasticity. In vivo OCE measures tissues in situ in their natural physiological condition and therefore provides greater accuracy and repeatability.

It is worth noting that while Eq. (1) used to estimate the tissue stiffness is strictly valid only for semi-infinite, homogeneous, isotropic and non-dissipative media, our results and theoretical analysis showed that it is a reasonable approximation for the human cornea in the frequency regime around 10 kHz. As was discussed earlier, the wavelength is sufficiently short compared to corneal thickness and curvature that the dispersion curve has nearly reached its asymptotic value (Supplementary Fig. S1). At scales comparable to the wavelength, the cornea is relatively uniform and tissue heterogeneity at subwavelength scales is averaged out into effective medium properties. Anisotropy, in general, results in different surface wave velocities depending on propagation direction, but not in the case of transverse isotropy when the principal axis is parallel to the surface normal. Finally, energy dissipation results in dispersion of the wave velocity as a function of frequency, an effect that combines with waveguide dispersion. This effect was shown to be significant in ex vivo porcine corneas^26^, but the dispersion curves of in vivo human corneas obtained here (Fig. 3d,f and Fig. S3) were relatively flat at high frequencies. This allows us to identify the high-frequency limit of the wave velocity as a Rayleigh wave and directly compute the material properties, without requiring the more complex analysis described in our previous work^26^.

Our data acquisition protocol using pure tone stimuli was well suited for in vivo OCE with limited measurement time. One key difference between our approach and some other proposed OCE techniques is the emphasis on frequency resolution over spatial resolution. The spatial-frequency analysis allowed us to distinguish Rayleigh-type waves from other spurious mechanical waves. At the pilot study stage, we favoured this approach to obtain deeper insight into the complex biophysical phenomena involved in corneal biomechanics. Interestingly, we found that the magnitude of spurious fast waves was less significant in human subjects compared to ex vivo porcine eyes^26^. This may be due to the difference in eyeball boundary conditions between the in vivo situation (orbital fat and muscles) and the experimental setup using a rigid eye holder. Given the reduced dispersion and relatively low spurious wave interference that we observed, improved spatial mapping within the same measurement time could reasonably be achieved by reducing the number of stimulus frequencies used.

Since the wave speed was extracted from measurements of the elastic wavelength, the spatial resolution of this OCE implementation is approximately equal to 1–2 wavelengths, which is as fine as 0.5–1 mm in the cornea and 1.5–3 mm in the sclera at an excitation frequency of 16 kHz. High-resolution spatial mapping is highly desirable for detecting focal biomechanical changes such as those observed in keratoconus. Although this is achievable by a series direct contacts, a steerable non-contact stimulus would be desirable, for example based on acoustic radiation force^23^. The current device configuration can be applicable to subjects with relatively normal corneas, such as pre-LASIK or *forme fruste* keratoconus subjects. However, for patients with mechanically weak corneas, the current contact probe would not be appropriate because of the safety concern. In such case, a non-contact mechanical excitation method based on acoustic radiation force driven at high repetition rate (> 2 kHz) may have potential as a viable approach. Another interesting alternative is the use of ambient noise or multiple decorrelated sources together with time-reversal retrieval algorithms^24^. The contact-based stimulation we used could potentially induce local stress, modifying the corneal curvature and perturbing wave propagation. This potential limitation was mitigated by using the smallest contact force possible while still ensuring proper mechanical contact, and by calculating wave propagation velocity in curvilinear coordinates. Furthermore, the spring-loaded tip improved the consistency of contact force across subjects, such that relative comparisons between subjects should remain valid.

This human study yielded a rather surprising correlation of shear modulus with age. Previous studies on the age-dependence of corneal biomechanics^7,27^ found a significant increase in the tensile modulus with age. For example, Knox et al. reported^27^ that the Young’s modulus of the cornea increased with age, approximately doubling from 0.27 MPa at age 20 (95% confidence interval, 0.22–0.31) to 0.52 (0.50–0.54) MPa at age 100. By contrast, our measurement of shear modulus indicates the opposite: the shear modulus decreases with age (Supplementary Fig. S5). Assuming both measurements are accurate, this discrepancy indicates that the in-plane shear modulus and the tensile Young's modulus are affected in different ways by aging. Considering the anisotropy of corneal tissue, it is not completely unexpected that different mechanical properties could be affected in different ways by aging. The Rayleigh-type wave velocity measured by our OCE technique primarily reflects the properties of the interfibrillar and interlamellar matrix. This modulus can be very different from the in-plane tensile rigidity measured by stretch or inflation testing, which reflects the stiffness of the collagen fibrils themselves (see Supplementary Note 1).

The age dependence of corneal shear modulus has not previously been examined, to the best of our knowledge. Interlamellar cohesive strength was studied by measuring the force required to separate two layers^36^. Correlation with age was poor but positive. However, the "peeling" measurement approach is likely to be dominated once again by tensile properties, more specifically the yield strength of the fibrils that interweave between lamellae. Overall, there is insufficient literature to either support or invalidate our hypothesis that shear cohesion decreases with age. Further studies using standard shear rheometry should be conducted, but this lies outside the scope of this article.

Collagen fibrils in the corneal stroma are not straight but crimped. As the tissue is strained along the plane by an external force, the collagen fibrils are stretched, and their Young’s modulus increases, making the tissue elastically nonlinear. It is expected that a higher IOP causes higher tension and thus Young’s modulus (Supplementary Fig. S6). However, our measurement indicates that corneal shear modulus is not correlated with IOP over a range of 13–18 mmHg. Given previous measurements of ex vivo eyeballs using inflation testing that showed increasing Young’s modulus with IOP^7^, our result is rather interesting (see Supplementary Note 2). One plausible explanation is that while ex vivo inflation causes instant elastic deformation of collagen fibrils, corneas in vivo have become adapted to the specific IOP level of each individual via remodelling, and that homeostasis is achieved at an optimal modulus that is more or less the same among healthy individuals. This hypothesis could potentially be tested by modulating the IOP rapidly by compression.

Our observations of age-related reduction in shear modulus and IOP independence warrant further investigation and verification in future studies. If confirmed, they could have important implications. According to our model, the mechanical properties of the matrix are primarily responsible for the flexibility of the cornea, which gives the tissue strength and toughness to withstand environmental damage. Cell–matrix interactions are also affected by the mechanical properties of the tissue, and can influence cell migration, tissue repair, and remodelling after surgery^37,38^. Finally, keratoconus involves changes in interlamellar cross-links and abnormal shear behaviour^39^. The shear-mechanical information obtained by Rayleigh-wave OCE could therefore provide valuable information for diagnosis and management of corneal ectasias.

## Methods

### Study design

A total of 12 healthy volunteers (7 males and 5 females) were recruited through Massachusetts General Hospital. This study followed a protocol approved by Partners Healthcare Institutional Review Board (IRB) and in accordance with the principles of the Declaration of Helsinki. Written informed consent was obtained from each subject after explanation of the nature and possible consequences of the study. The age of the subjects ranged from 25 to 67 years. Individuals with a refractive error beyond ± 6 dioptres, history of prior refractive eye surgery such as LASIK or cataract removal surgery, corneal surface disorders such as dry eye, history of glaucoma or diabetes, and/or significant eye disease were excluded from the study. IOP values were within normal range for all subjects. OCE was performed in the left eye only for each subject. Geometric parameters, such as CCT and corneal curvature, were extracted from volumetric OCT scan images of the anterior segment, obtained using the same OCT system. Tonometry and slit lamp examination with fluorescein stain were performed by an optometrist approximately 15–45 min after the OCE measurements to record IOP and examine epithelial status.

### Instrument design

The instrument was modified from the system previously described^25,26^. Briefly, it comprises a home-built, polygon-swept laser source (centre wavelength: 1300 nm, bandwidth: 108 nm, sweep rate (A-line rate): 43.1 kHz, illumination power on the cornea: 15 mW, in compliance with the ANSI-Z136.1-2014 safety standard), a 2-axis galvanometer scanner (Cambridge Technology, 6210H), and an objective lens (Thorlabs, LSM54-1310, working distance: 64 mm, transverse resolution: 30 μm). A human interface modified from a slit-lamp instrument has a chin and forehead rest and a joystick for coarse manual alignment. A multi-purpose input/output board (USB-6353, National Instruments) was used to generate analog waveforms to operate the beam scanner and the mechanical probe. The waveforms were synchronized to the wavelength sweep cycles of the laser and data acquisition clocks, ensuring phase-synchronous operation. The probe tip was fabricated by 3D printing of biocompatible material (Formlabs, Dental SG) to a spherical shape with a diameter of 2 mm and was hand-polished to minimize surface roughness. The tip was glued to a spring-loaded lever arm with a constant resistance force of ~ 20 mN. The lever arm was vibrated by the push–pull operation of a pair of electrodynamic transducers (Adafruit, 1784) that were positioned symmetrically with respect to a central pivot point. An audio amplifier (Crown Audio, XLI) was used to amplify the driving waveform to the transducers. The oscillating tip generated a waveform with frequency-dependent amplitude, but the particle velocity (time derivative of the displacement waveform) was kept approximatively constant and below 10 mm/s at the point of contact. The corresponding intensity for a wave traveling at 7.5 m/s is about 7.5 mW/cm^2^, which is below the FDA-recommended maximum of 17 mW/cm^2^ for ophthalmic applications^32^.

### Imaging protocol

Proparacaine ophthalmic drops were administered as a topical anaesthetic to the targeted eye prior to OCE and tonometry. With the probe tip away from the eye, the subject’s head was placed on the human interface. The OCT beam was aligned to the eye using standard anatomic OCT imaging while the subject gazed at a fixation target positioned behind the instrument at locations appropriate for corneal, limbal, and scleral measurements, respectively. The subject was instructed to remain stationary and refrain from blinking as the probe tip was moved towards the cornea using a thumb screw until it made contact. With the probe tip in contact with the cornea, OCE data along the region of interest were taken. The tip was then withdrawn from the eye. This single measurement took ~ 10 s. Figure S2 depicts time diagrams for the data acquisition protocol. The M-B scan protocol comprised the acquisition of $M$ consecutive A-lines at each transverse location $x$ while a mechanical-frequency scan was completed. The OCT beam was then moved to the next location, and the stimulus waveform was repeated. Typically, we used $M$ = 172 at an A-line rate of 43 kHz, so each M-scan took 4 ms. We designed the beam-scanning pattern to sample $p$ = 8 points per elastic wavelength ($\lambda$), a factor of 4 above the Nyquist limit. The sampling interval $\delta x$ was varied with the vibration frequency ($f$) following $\delta x = V/(fp)$, where the wave speed $V$ ≈ 7.5 m/s for the human cornea. Considering the attenuation of the elastic waves, the total beam-scan range was set to 4 $\lambda$, so the total number of scan points $P$ was equal to 4 $p$ = 32. The measurement time for a single M-B scan per frequency was 0.128 s. A complete measurement included = $N$8 different frequencies (2–16 kHz by a step of 2 kHz), which took 1 s. This frequency scan was repeated 10 times ($N_{Rep}$). Therefore, the total measurement time was 10 s. Note that each M-B scan was processed independently. So, only the subject movement during each M-B scan time of 0.128 s could affect the data quality.

### Displacement field measurement

The raw data collected from the M-B scan was processed using standard swept-source phase-stabilized algorithms^25^. The resulting complex-valued OCT tomogram is denoted $A({\varvec{r}},\;t)$ where $t$ is the time and ${\varvec{r}} = (x, \;z)$ represents the transverse and axial position vector within the dataset. The axial component of the displacement field $u_{z} ({\varvec{r}},\;t)$ is computed from $A({\varvec{r}},\;t)$ as:

$${\Delta }\phi ({\varvec{r}},\;t_{i} ) = \arg \left( {\mathop \sum \limits_{{{\varvec{r}} {\text{in ROI}}}} A^{*} ({\varvec{r}},\;t_{i - 1} )A({\varvec{r}},\;t_{i} )} \right)$$

$$\phi ({\varvec{r}},\;t_{m} ) = \mathop \sum \limits_{i = 1}^{m} {\Delta }\phi ({\varvec{r}},\;t_{i} ) , \quad m = 1,2, \ldots M$$

$$u_{z} ({\varvec{r}},\;t_{m} ) = \frac{{\lambda_{0} }}{{4\pi n_{c} }}\left( {\phi ({\varvec{r}},\;t_{m} ) + \phi ({\varvec{r}}_{{{\text{top}}}} ,\;t_{m} )\frac{{n_{c} - n_{0} }}{{n_{0} }}} \right)$$

where ROI is a small neighbourhood around ***r*** (11 transverse and 1 axial points for z-axis projection), $\lambda_{0}$ = 1300 nm is the mean optical wavelength, $n_{0}$ = 1 and $n_{c}$ = 1.376 are the refractive indices of the air and the cornea, respectively, and $\phi ({\varvec{r}}_{{{\text{top}}}} ,\;t)$ is the optical phase measured at the air-tissue boundary^40^.

## Supplementary information

Supplementary Information.

## Data Availability

All data generated or analysed during this study are included in this published article and its Supplementary Information files.
