# Supplementary material for: In vivo measurement of shear modulus of the human cornea using optical coherence elastography
Source: Sci Rep. 2020 Oct 15;10:17366. doi: 10.1038/s41598-020-74383-4 (PMC7567833; doi:10.1038/s41598-020-74383-4)
Supplement: Supplementary file 1 — Supplementary Information. [file 41598_2020_74383_MOESM1_ESM.docx]

Supplementary Information

In Vivo Measurement of Shear Modulus of the Human Cornea Using Optical Coherence Elastography

Antoine Ramier,^1,2^ Amira M. Eltony,^1^ YiTong Chen,^1,3^ Fatima Clouser,^1^ Judith S. Birkenfeld,^1,4,5^ Amy Watts,^6^ Seok-Hyun Yun^1,2,*^

^1^ Wellman Center for photomedicine and Harvard Medical School, Massachusetts General Hospital, 50 Blossom St., Boston, MA, USA;

^2^ Harvard-MIT division of Health Sciences and Technology, Cambridge, MA, USA;

^3^ Department of Automation, Tsinghua University, Beijing 100084, China;

^4^ Research Laboratory of Electronics, Massachusetts Institute of Technology, 77 Massachusetts Ave, Cambridge, MA 02139, USA

^5^ Instituto de Optica (IO-CSIC), C/ Serrano, 121 Madrid, Spain;

^6^ Department of Ophthalmology, Massachusetts Eye and Ear, 243 Charles Street, Boston, MA, 02114, USA;

* Corresponding author: syun@mgh.harvard.edu

# SUPPLEMENTARY FIGURES

**Fig. S1.** Guided-wave propagation in a corneal flap-like plate. **a**, A method for exciting elastic waves in the cornea. **b**, Dispersion curves of elastic-wave modes in the structure with no in-plane tension. The dashed curve indicates the effective propagation speed $c/c_{s}$ (where $c$ is the phase velocity, and $c_{s}$ is the pure shear wave velocity) of the combined excited wave measured along the air-cornea surface. Circles indicate values at an excitation frequency $f$ of 10 kHz for a plate thickness $h$ of 500 μm, shear modulus $G_{zx}$ of 60 kPa and a density $\rho$ of 1.05 g/cm^3^. **c**, Coefficients of coupling to various Lamb-wave modes in the cornea by the contact vibrating probe. The amplitude coupling coefficients were calculated by using an overlap integral of the modal displacement field and the uniform vertical motion generated by the external stress. At acoustic frequencies below 5 kHz, it is the lowest-order flexural, quasi-asymmetric mode (A0­) that is predominantly excited. For higher frequencies in the range of 10-16 kHz, both the A0­ and the lowest symmetric mode (S0), as well as the A1 mode, are excited. **d**, Displacement profiles for the S0 and A0 modes and a superposition of the modes with calculated coupling coefficients at 10 kHz.

**Fig. S2. Timing diagrams of the OCE system.** The sinusoidal waveform was applied to the transducers in the contact probe and step-tuned over $N$ frequencies from 2 to 16 kHz. For each frequency, vibrography data at P transverse locations in the tissue was acquired, with $M$ A-lines per point. This M-B scan was repeated by $N_{rep}$ times.

**Fig. S3. Measured elastic-wave velocity of the human cornea for 12 subjects.** The box-whisker data representation corresponds to the quartiles and min-max of 10 frequency-scan data. Solid lines (red) represent the median of the data in a range from 6 to 16 kHz, and dashed lines indicate 95% confidence interval. Greyed-out boxes correspond to frequencies that were left out of the analysis due to interference with spurious waves or insufficient wave amplitude.

**Fig. S4. Measured elastic-wave attenuation distance of the human cornea for 12 subjects.** The box-whisker data representation corresponds to the quartiles and min-max of 10 frequency-scan data. Solid lines (red) represent curve fits to the data in a range from 6 to 16 kHz. Greyed-out boxes correspond to frequencies that were left out of the analysis due to interference with spurious waves or insufficient wave amplitude.

**Fig. S5. Comparison of shear modulus obtained *in vivo* in this work to previously published *ex vivo* tensile modulus data for human corneas.** Blue circles: experimental data; dashed lines: prediction interval limits; Cyan solid line: linear regression of the shear modulus data calculated from the wave speeds shown in Fig. 6 (B). Magenta solid line: linear regression curve of Young’s modulus data obtained with cadaver tissues by Knox *et al*. [from Fig. 4 in Ref. 35]. The two parameters are plotted with a scale of 1:3 ($G\approx E/3)$. Dashed lines: prediction intervals. Note that the opposite age-dependent trends.


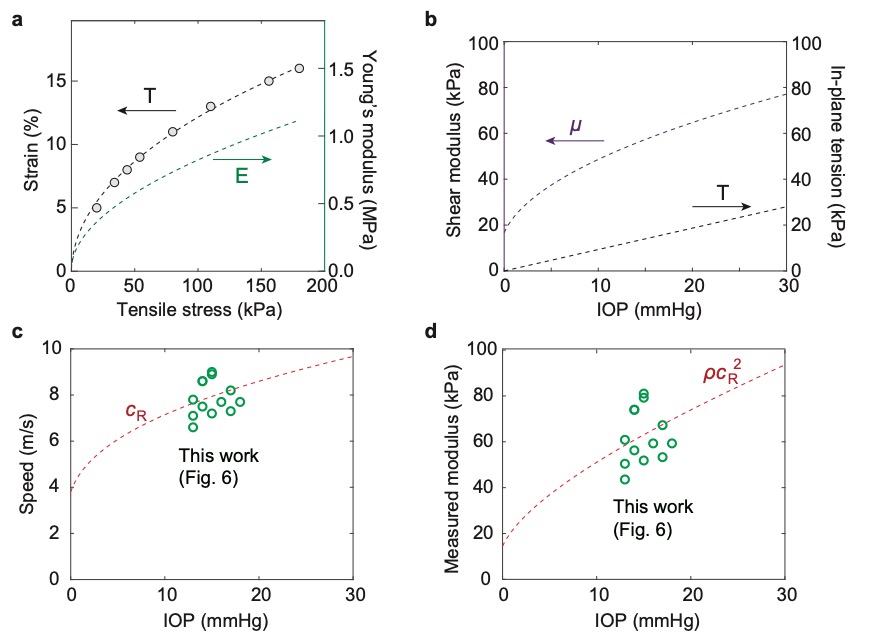


**Fig. S6. Estimation of the effect of IOP on corneal shear modulus.** **a**, Tensile stress–strain relationship of the human cornea derived from the *ex vivo* extensometry data obtained by Wollensak *et al*. (See Supplementary Note 2). Circles: experimental data; black dashed line: curve fit assuming that the stress is a second-order polynomial function of strain. Green curve: Young’s modulus ($E_{xx})$ calculated from the strain-stress curve fit. **b**, Shear modulus $G_{xz}=aE_{xx}$, where $a=$ 1/6 was chosen to fit the shear modulus to the measurement data in Fig. 6d. The coefficient $a$ should be 1/3 for homogeneous materials but could be smaller than 1/3 for the corneal tissues owing to the transverse anisotropy of the lamellar structure (see Supplementary Note 1). Right y-axis shows calculated in-plane tension on the corneal tissue as a function of IOP (See Supplementary Note 2.) **c**, Theoretical values for elastic wave speed (dashed lines) and the *in vivo* data (circles; each circle represents the mean value from a healthy subject) obtained in this work. **d**, Theoretical curve for measured shear modulus from the Rayleigh wave speed (dashed line) and the data (circles) calculated from the *in vivo* data assuming a constant density for all subjects.

# Supplementary note S1. Mechanical model of the cornea

From X-ray diffraction data, the average fibril diameter for human corneas is 30.8 nm and the interfibrillar spacing is 55.3 nm^1^. The volume fraction of collagen fibrils is estimated to be $v_{f}\approx0.22$.

The rest of the volume is occupied by extrafibrillar tissue, largely comprising water (~78%) and non-fibrillar proteins. We refer to this tissue as a hydrogel.

For simplicity in modeling, each collagen fibril is approximated by a square rod with the same cross-sectional area^2^. The structure of a single lamella can be viewed as a stack of two horizontal layers, Layer-1 that contains fibers and Layer-2 between Layer-1’s. Let $v_{1}$ and $v_{2}$ denote the volume fractions of the layers ($v_{1}+v_{2}=1)$.

The Layer-1 consists of collagen fibers and interfibrillar tissue, each occupying volume fractions of $v_{1}^{(f)}$ and $v_{1}^{(g)}$, respectively, where $(f)$ indicates collagen fibrils and $(g)$ interstitial hydrogels and $v_{1}^{(f)} +v_{1}^{(g)} =1$. This is illustrated in Fig. 1c. For the hexagonal lattice, $v_{1}^{\left( g \right)}/v_{1}^{\left( f \right)}={(v_{f}\sqrt{3}/2)}^{-0.5}-1\approx1.29$ for $v_{f}=0.22$. So, $v_{1}^{\left( f \right)}\approx0.45$ and $v_{1}^{\left( g \right)}\approx0.55$. We also find that $v_{2}/v_{1}=(\sqrt{3}/2-1)v_{1}^{\left( f \right)}/v_{1}^{\left( g \right)}+\sqrt{3}/2\approx0.76$. So, $v_{1}\approx0.57$ and $v_{2}\approx0.43$.

## Shear modulus

We define a local coordinate system for each lamella, in which the 1-axis is along the orientation of collagen fibrils.

Let $\sigma_{ij}^{(m)}$ and $\varepsilon_{ij}^{(m)}$ denote the $ij$-th matrix element of the stress and strain matrices, respectively, for material $m$. By symmetry, we consider each material has the hexagonal symmetry. Later we will consider isotropic hydrogels. The Hooke’s law relation for shear motion can be expressed with a diagonal matrix of shear moduli $G_{ij}^{(m)}$:

$$\left( \begin{matrix} \sigma_{23}^{(m)} \\ \sigma_{13}^{(m)} \\ \sigma_{12}^{(m)} \end{matrix} \right)=\left( \begin{matrix} G_{23}^{(m)} & 0 & 0 \\ 0 & G_{13}^{(m)} & 0 \\ 0 & 0 & G_{12}^{(m)} \end{matrix} \right)\left( \begin{matrix} \varepsilon_{23}^{(m)} \\ \varepsilon_{13}^{(m)} \\ \varepsilon_{12}^{(m)} \end{matrix} \right)$$

By symmetry, $G_{13}^{(m)}=G_{12}^{(m)}$.

Let us consider Layer-1 under shear stress in the 23 direction, we find that the shear stress and strain of Layer-1 satisfy the following equations:

$$\sigma_{23}^{(1)}=\sigma_{23}^{\left( f \right)}=\sigma_{23}^{(g)}$$

$$\varepsilon_{23}^{(1)}=\varepsilon_{23}^{\left( f \right)}v_{1}^{\left( f \right)}+\varepsilon_{23}^{(g)}v_{1}^{\left( g \right)}$$

These equations lead to

$$\frac{1}{G_{23}^{(1)}}=\frac{v_{1}^{\left( f \right)}}{G_{23}^{(f)}}+\frac{v_{1}^{\left( g \right)}}{G_{23}^{(g)}}$$

Since $G_{23}^{(f)}\gg G_{23}^{\left( g \right)}$, we find $G_{23}^{(1)}\approx G_{23}^{\left( g \right)}/v_{1}^{\left( g \right)}$.

Likewise, for the other out-of-plane shear modulus in the 12 direction we find

$$G_{12}^{(1)}\approx G_{12}^{\left( g \right)}/v_{1}^{\left( g \right)}$$

For in-plane shear stress in the 13 direction, we find

$$\sigma_{13}^{(1)}=\sigma_{13}^{\left( f \right)}v_{1}^{\left( f \right)}+\sigma_{13}^{(g)}v_{1}^{\left( g \right)}$$

$$\varepsilon_{13}^{(1)}=\varepsilon_{13}^{\left( f \right)}=\varepsilon_{13}^{(g)}$$

$$G_{13}^{(1)}=G_{13}^{(f)}v_{1}^{\left( f \right)}+G_{13}^{(g)}v_{1}^{\left( g \right)}\approx G_{13}^{(f)}v_{1}^{\left( f \right)}$$

Now, consider Layer-2 that is connected with Layer-1 along the 3-axis. Now, shear involving the 3-axis is out-of-plane shear, and shear in the 12 direction is in-plane shear. The shear moduli of a single lamella, denoted by superscript $(l)$, are given by:

$$\frac{1}{G_{23}^{(l)}}=\frac{v_{1}}{G_{23}^{(1)}}+\frac{v_{2}}{G_{23}^{(2)}}$$

$$\frac{1}{G_{13}^{(l)}}=\frac{v_{1}}{G_{13}^{(1)}}+\frac{v_{2}}{G_{13}^{(2)}}$$

$$G_{12}^{(l)}=G_{12}^{(1)}v_{1}+G_{12}^{(2)}v_{2}$$

Using $G_{ij}^{(2)}=G_{ij}^{\left( g \right)}$ and $G_{ij}^{(f)}\gg G_{ij}^{\left( g \right)}$, we get

$$G_{23}^{(l)}\approx G_{23}^{\left( g \right)}/(v_{1}^{\left( g \right)}v_{1}+v_{2})\approx1.34 G_{23}^{\left( g \right)}$$

$$G_{13}^{(l)}\approx G_{13}^{\left( g \right)}/v_{2}\approx2.32 G_{13}^{\left( g \right)}$$

$$G_{12}^{(l)}=G_{12}^{\left( g \right)}(v_{1}/v_{1}^{\left( g \right)}+v_{2}) \approx1.47 G_{12}^{\left( g \right)}$$

Note that the all three shear stiffness components of the lamella are governed by the shear modulus of the extrafibrillar hydrogel. The contribution of much stiffer collagen fibers is quenched by the surrounding softer gel.

Because of the hexagonal symmetry, $G_{12}^{(l)}=G_{13}^{(l)}$ is expected. The discrepancy in the above result is due to the approximation in the model. This small error is reduced by averaging of the two values. For the case of isotropic extrafibrillar gels with $\mu=G_{ij}^{(g)}$, we get $G_{12}^{(l)}=G_{13}^{(l)}\approx1.9 \mu$.

Finally, we consider the full lamellar structure, in which lamellae with orthogonal orientations are stacked along the z-axis (Fig. 1c). The total shear stiffness of the corneal tissue is as follows:

$$G_{yz}^{(tot)}=G_{xz}^{(tot)}=\frac{2G_{23}^{(l)}G_{13}^{(l)}}{G_{23}^{(l)}+G_{13}^{(l)}}\approx1.6 \mu$$

$$G_{xy}^{(tot)}=0.5 G_{12}^{(l)}+0.5 G_{12}^{(l)}=G_{12}^{(l)}\approx1.9 \mu$$

## Young’s modulus (tensile test)

A tensile test is conducted by applying tensile stress to corneal tissues. For linear tension along the 2-axis, where $\sigma_{22}\neq0$ and $\sigma_{11}=\sigma_{33}=0$, the Hooke’s law equation states

$$\sigma_{22}^{(m)}=E_{22}^{(m)}\varepsilon_{22}^{(m)}$$

where $E_{22}^{(m)}$ is Young’s modulus along the 2-axis.

For Layer-1, we find:

$$\sigma_{22}^{(1)}=\sigma_{22}^{\left( f \right)}=\sigma_{22}^{(g)}$$

$\varepsilon_{22}^{(1)}=\varepsilon_{22}^{\left( f \right)}v_{1}^{\left( f \right)}+\varepsilon_{22}^{(g)}v_{1}^{\left( g \right)}$

$$\frac{1}{E_{22}^{(1)}}=\frac{v_{1}^{\left( f \right)}}{E_{22}^{(f)}}+\frac{v_{1}^{\left( g \right)}}{E_{22}^{(g)}}$$

The reported values in literature for $E_{ii}^{(f)}$, typically obtained using AFM nanoindentation for hydrated collagen microfibrils^3^, are on the order of 500 MPa. Typically, $E_{ii}^{\left( g \right)}\approx3\mu$ and are in the order of magnitude of 50 kPa. Since $E_{ii}^{(f)}\gg E_{ii}^{\left( g \right)}$, we get $E_{22}^{(1)}\approx E_{22}^{\left( g \right)}/v_{1}^{\left( g \right)}$.

For in-plane tension along the 11 direction, we find

$$\sigma_{11}^{(1)}=\sigma_{11}^{\left( f \right)}v_{1}^{\left( f \right)}+\sigma_{11}^{(g)}v_{1}^{\left( g \right)}$$

$$\varepsilon_{11}^{(1)}=\varepsilon_{11}^{\left( f \right)}=\varepsilon_{11}^{(g)}$$

$$E_{11}^{(1)}=E_{11}^{(f)}v_{1}^{\left( f \right)}+E_{11}^{(g)}v_{1}^{\left( g \right)}\approx E_{11}^{(f)}v_{1}^{\left( f \right)}$$

Likewise,

$$E_{33}^{(1)}=E_{33}^{(f)}v_{1}^{\left( f \right)}+E_{33}^{(g)}v_{1}^{\left( g \right)}\approx E_{33}^{(f)}v_{1}^{\left( f \right)}$$

The tensile stiffness of a single lamella is as follows:

$$E_{11}^{(l)}=E_{11}^{(1)}v_{1}+E_{11}^{(g)}v_{2}\approx E_{11}^{\left( f \right)}v_{1}^{\left( f \right)}v_{1}\approx0.26 E_{11}^{\left( f \right)}$$

$$E_{22}^{(l)}=E_{22}^{(1)}v_{1}+E_{22}^{(g)}v_{2}\approx E_{22}^{\left( g \right)}(v_{1}/v_{1}^{\left( g \right)}+v_{2}) \approx1.47 E_{22}^{(g)}$$

$$E_{33}^{(l)}=\frac{E_{33}^{(1)}E_{33}^{(g)}}{E_{33}^{(g)}v_{1}+E_{33}^{(1)}v_{2}}\approx E_{33}^{\left( g \right)}/v_{2}\approx2.32 E_{33}^{\left( g \right)}$$

By symmetry consideration, we average $E_{22}^{(l)}=E_{33}^{(l)}$ to obtain $E_{22}^{(l)}=E_{33}^{(l)}\approx5.7 \mu$.

For the corneal tissue consisting of lamellae stacked with orthogonal orientations, we find

$$E_{xx}^{(tot)}=E_{yy}^{(tot)}=0.5 E_{11}^{(l)}+0.5 E_{22}^{(l)}\approx0.13 E_{11}^{\left( f \right)}$$

$$E_{zz}^{(tot)}=E_{33}^{(l)}\approx5.7 \mu$$

The in-plane Young’s modulus is governed by the tensile modulus of the fibrils.

It is worth considering a case where the corneal tissue consists of lamellae with random orientations instead of two orthogonal orientations. For lamellae with their fibrillar orientations are tilt from the direction of tension, shear stress is also applied to the fibrillar axis in addition to tensile stress. These lamellae would exhibit low stiffness, at the level similar to that of the hydrogel. Only a small fraction of lamellae with fibrillar orientations within a small angle, $\sim G_{13}^{\left( l \right)}/E_{11}^{\left( l \right)}$, give fibril-supported, high stiffness. The overall tensile stiffness should be in the order of the tensile strength of the hydrogel. This random lamella structure is transverse isotropic and satisfies the condition that $E_{xx}^{(tot)}=E_{yy}^{(tot)}=3G_{xy}^{(tot)}\approx5.7 \mu$.

In human corneas, the lamellar orientations are not totally random. They are dominantly aligned along the horizontal and vertical axes of the eye, especially near the corneal center. Therefore, the in-plane tensile modulus is largely, although not entirely, governed by the stiffer collagen fibrils rather than extrafibrillar gels.

## Propagation of Rayleigh waves versus tensile test

The propagation speed of Rayleigh elastic waves propagating along the corneal plane is governed by $G_{yz}^{(tot)}\approx1.6 \mu$. Therefore, Rayleigh-wave OCE reveals the elasticity of the extrafibrillar tissue.

When the stroma has two dominant lamellar orientations, the tensile test measures in-plane Young’s modulus, which is $0.13 E_{11}^{\left( f \right)}$. Therefore, extensometry reveals the axial tensile modulus of collagen fibrils.

# Supplementary note S2. The effect of intraocular pressure on shear modulus

The IOP affects OCE measurement in two ways. First, the corneal tissue is a nonlinear material with its stiffness increasing with strain. As collagen fibers are stretched, more tension is required to generate the same amount of strain. As a result, the shear modulus value of the tissue varies with IOP. Second, tension affects the propagation of elastic waves along the cornea. We will discuss it in more detail below.

It is important to understand the material and waveguide effects of corneal tension on the mechanical wave propagation quantitatively. This has not been studied in the context of OCE. Tension stretches the tissue and generates strain $\varepsilon$. We write this relation as $T=E \varepsilon$, where $E$ is effective Young’s modulus ($E_{xx}$). As the collagen fibers in the tissue are stretched by the tension, the shear modulus of the tissue increases. To obtain the strain-stress information, we use the data obtained by Wollensak *et al*.^4^ (Circles in Fig. S6a). Adjusting the data for zero stain at zero stress, we fitted the data using two coupled equations to get $E \left( kPa \right)\approx100*(1+60 \varepsilon)$ and $T=E*\varepsilon$.

Pressure-induced tensile stress in the corneal wall can be calculated using Young-Laplace’s equation, $T=IOP*\left( R/2h \right)$. For corneas with a radius of curvature of 7.5 mm ($R$), and a thickness of 0.54 mm ($h$), the stress is $T\approx7*IOP$. For the normal range of IOP from 12 to 21 mmHg (1 kPa $\approx$ 7.5 mmHg), we estimate: T = 11 to 20 kPa and $\varepsilon$ = 3.5 to 5% (Fig. S6b).

Assume the cornea being a thin plate with a thickness $h$ and shear modulus $\mu$ under tensile stress $T$. Using the standard thin plate theory by Timoshenko^5^, we write the following equation for a pure bending wave: $\rho h\frac{d^{2}w}{dt^{2}}=-D\frac{d^{4}w}{dx^{4}}+Th\frac{d^{2}w}{dx^{2}}$, where $D=\mu h^{3}/3$ is bending modulus. Putting $w=cos(kx-\omega t)$, we find $\rho h\omega^{2}=Dk^{4}+Thk^{2}$, and this leads to $\omega/k=\sqrt{(\mu/3\rho)({kh)}^{2}+T/\rho}$. This expression is valid at quasi static condition, which is equivalent to an elastic wavelength substantially larger than the plate thickness (i.e. $kh\ll1$) so that deformation is close to pure bending. This regime does not hold for high frequencies at which the acoustic wavelength is shorter than $h$. At this high frequency, the displacement energy is localized along the surface. Since the first term in the square root corresponds to the elastic speed without tension and we know that $c_{R}\approx0.94 c_{s}$, we may estimate:

$$c_{R}\approx\sqrt{0.91 \mu/\rho+T/\rho}$$

Note that $\mu$ is a function of $T$ because of the nonlinear elasticity of corneal tissues. For corneal tissues, the precise relation between effective Young’s modulus $E$ and effective shear modulus $\mu$, or $G_{xz}$, would vary depending on specific arrangement of lamella and their angle to the tensile stress. To fit the human data, we use $\mu=6.7*\left( 1+60 \varepsilon\right)$ kPa and calculate the magnitude of $c_{s}$ and $c_{R}$ as a function of IOP (Fig. S6c). The *in vivo* experimental data (Fig. 6d) have considerable interpersonal variations. We did not find a statistical correlation between the measurement data and IOP (Fig. S6d).

One way to verify whether the cornea follows the IOP dependence expected from the nonlinear elasticity of the tissue *in vivo* would be measuring shear-wave speed from a subject over time as the IOP is modulated by either a physical or chemical method. The corneal tissue in a specific subject may undergo remodeling or plastic change in response to its mean IOP level. It will be interesting to investigate this possibility.

# Supplementary references

1. Meek, K. M. & Boote, C. The organization of collagen in the corneal stroma. *Exp. Eye Res.* **78**, 503–512 (2004).

2. Nayfeh, A. H. *Wave Propagation in Layered Anisotropic Media : With Applications to Composites*. (Elsevier, 1995).

3. Gautieri, A., Vesentini, S., Redaelli, A. & Buehler, M. J. Hierarchical Structure and Nanomechanics of Collagen Microfibrils from the Atomistic Scale Up. *Nano Lett.* **11**, 757–766 (2011).

4. Wollensak, G., Spoerl, E. & Seiler, T. Stress-strain measurements of human and porcine corneas after riboflavin–ultraviolet-A-induced cross-linking. *J. Cataract Refract. Surg.* **29**, 1780–1785 (2003).

5. Timoshenko, S. P. & Gere, J. M. *Theory of Elastic Stability*. (Dover Publications, 2009).
